# Electromechanical Characteristics Analysis of Magnetic Shield on Superconducting Magnetic Levitation Train

**DOI:** 10.3390/mi16111248

**Published:** 2025-10-31

**Authors:** Mingyuan Hu, Lei Zhang, Ran Tao, Ping Wang

**Affiliations:** 1 School of Electrical Engineering, Shandong Huayu University of Technology, Dezhou 253000, China; wp@huayu.edu.cn; 2 School of Electrical Engineering, Beijing Jiaotong University, Beijing 100091, China; 22110464@bjtu.edu.cn; 3 School of Electrical Engineering, Southeast University, Nanjing 210096, China; cumt_ran@163.com

**Keywords:** superconducting maglev system, magnetic shield, magnetic flux density

## Abstract

The guest room and aisle of electric high-speed maglev train must be shielded from leakage magnetic flux produced by superconducting strong magnetic field. To reduce magnetic leakage, the superconducting magnetic levitation system structure is obtained by extended lagrangian optimization method. The optimized superconducting coil structure has the advantages of reducing magnetic leakage, improving magnetic field utilization, reducing the weight of the magnetic isolation plate and the weight of the maglev train, and enhancing the load-bearing capacity of the maglev train. Based on optimized superconducting coil parameters for high-speed maglev, the magnetic shielding effect at the aisle and the guest room, the magnetic flux density distribution at the magnetic shielding is calculated and analyzed through analytical calculation. The relevant conclusions indicate that the magnetic suspension structure has the advantages of reducing end coil leakage flux and the weight of the high-speed maglev train.

## 1. Introduction

Superconducting (SC) coils play an extremely important role in the propulsion, levitation, guidance, damping, braking, and harmonic generation of high-speed ground transportation [1,2]. Meanwhile, it is quite economical to use SC coils as excitation coils, as an SC coil can easily provide a stronger magnetic field than a conventional permanent magnet [3,4]. Therefore, the levitation and guidance system, linear synchronous motor (LSM) propulsion system, harmonic power generation system, and damping brake system, with SC coils as excitation source, have been fully and deeply researched.

Ma et al. proposed a novel calculation method to calculate the magnetic field and three-dimensional (3D) forces of air-cored LSM for SC electrodynamic suspension (EDS) train [5,6]. Kou et al. proposed a harmonic magnetic field analysis method of double-sided air-cored SC LSM for EDS train, and the 3D electromagnetic force characteristics are analyzed in detail [7,8]. Deng et al. established a 3D transient numerical model to discuss the dynamic electromagnetic characteristics of SC EDS train [9]. A. Morini et al. analyzed the propulsion, levitation, drag, and lateral forces acting on EDS-MAGLEV trains [10,11]. Y-D Chung et al. described the conceptual design of a high-temperature SC receiver coil in the wireless power charging system for an SC maglev train [12]. Matsue et al. developed a linear generator system for auxiliary power of the SC maglev train [13]. However, the above SC magnetic suspension system has the disadvantages of a complex electromagnetic structure, high investment, and maintenance costs. To accelerate the commercial operation of the SC magnetic levitation system, an integrated propulsion, levitation, and guidance (PLG) system is presented. Fujie proposed the PLG method for a more favorable SC maglev ground coil system [14]. Ohashi introduced the passive and the active damper coil system to increase the damping of the SC levitation system [15]. He et al. discussed magnetic damping forces in a figure-eight-shaped coil suspension system [16].

Oh et al. deal with the simplex optimization of a shield coil geometry for improving the magnetic shielding performance of air-core SC generators [17]. However, the harm of SC strong magnetic fields has not been further discussed. Further, a magnetic shield against the magnetic field produced by SC coils is also an important task, especially for reducing the leakage magnetic flux generated by the SC coils, which would reduce the amount of necessary magnetic shielding material and improve levitation-drag-ratio performance. Andrioll et al. presented a method to optimize the geometrical size of both the SC and levitation coils, with the aim of minimizing the field harmonic content on the shield [18]. Pamidi et al. presented magnetic shielding characteristics of hybrid shield materials [19]. Sugoushi et al. observed the influence of the SC magnetic shield on the electromagnetic properties of SC synchronous motors for a turbo-electric propulsion system [20]. Li et al. presented and designed a uniform magnetic field coil with a magnetic shield [21]. The shielding performances of ac magnetics field generated by the propulsion windings for the case of an LSM active guideway are discussed [22].

By utilizing the mapping relationship between the SC coil structure and magnetic shielding characteristics, the extended lagrangian method was used to optimize the SC coil structure. Firstly, the SC coils and magnetic shielding structure are introduced. Then, based on electromagnetic field characteristics and optimization theory, the electromagnetic optimization of the shield plate is derived, and the SC coil size is obtained. Finally, the magnetic shielding effect at the aisle and the guest room, the magnetic flux density distribution at the shield plate characteristics are calculated and analyzed through analytical calculation. The relevant conclusions indicate that the structure has the advantages of reducing the end coil leakage flux and the weight of the high-speed maglev train.

## 2. The SC Coils and Magnetic Shield Structure

**Figure 1:**
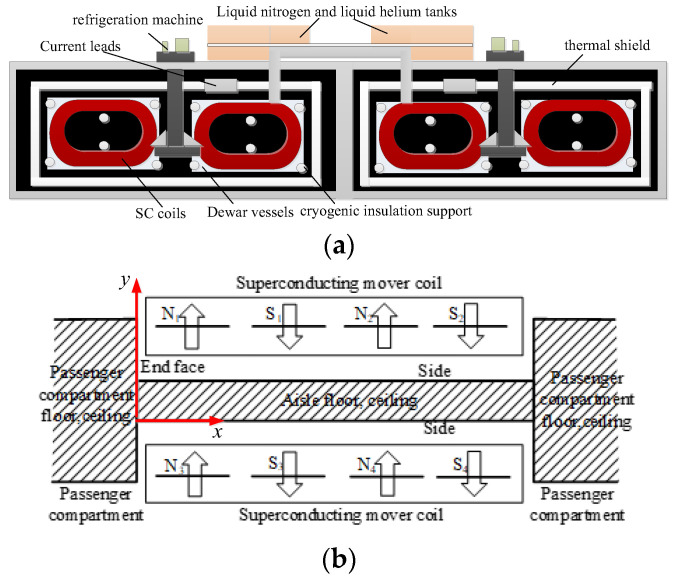
The structure of cooling facilities, SC coils, and magnetic shield (**a**) the cooling facilities, (**b**) the SC coils and magnetic shield.

The SC maglev system relies on the electromagnetic field generated by the SC coils for propulsion, levitation, guidance, power generation, and damping braking. The function of the SC coils is significant, and it has strong magnetic field characteristics, so the leakage magnetic field generated by the SC coils must be shielded in the guest room. The SC coils adopt Nb-Ti low-temperature SC wire, with a geometric shape of a track-shaped double cake winding, as shown in Figure 1a. The main basic cooling facilities for SC coils include on-board helium and nitrogen refrigerators, liquid nitrogen and liquid helium, helium buffer tanks, helium compressors, external Dewar vessels, insulation supports, cold screens, and cooling pipes. The design of this cooling system enables the SC coils to operate stably in a 4.2 K environment. The compact low-temperature container layout and modular refrigeration unit ensure the rationality of the spatial design of cooling infrastructure, SC coils, and shielding systems. The SC coils are installed on the articulated bogie; the magnetic shield plate made of industrial pure steel shall be installed outside the guest room. Generally speaking, in order to reduce the impact of a strong magnetic field on the passengers in the guest room, the peak magnetic field target can be set as 5 mT [22]. The structure of SC coils and the magnetic shield is shown in Figure 1b, presented in the form of a vertical view. The shield plates are installed in the shaded area. The two guest rooms are connected through the aisle, which is higher in height than the guest room, to keep a distance from the SC coils. The shield plates are only installed in the area around the articulated bogie. The SC coils parameters before optimization are shown in Table 1. To reduce the harm of magnetic fields generated by SC coils to the human body, the shape of SC coils should be optimized.

## 3. The Optimization of Maglev SC Coils

Firstly, to simplify the analysis of magnetic shielding theory, the following assumptions are made [23]:

(1) The magnetic permeability of the magnetic shielding plate is infinite
(2) The internal area that needs to be shielded is completely covered by a magnetic shielding plate
(3) The magnetic shielding plate cannot carry magnetic flux higher than the saturation magnetic flux density *B_s_*

**Figure 2:**
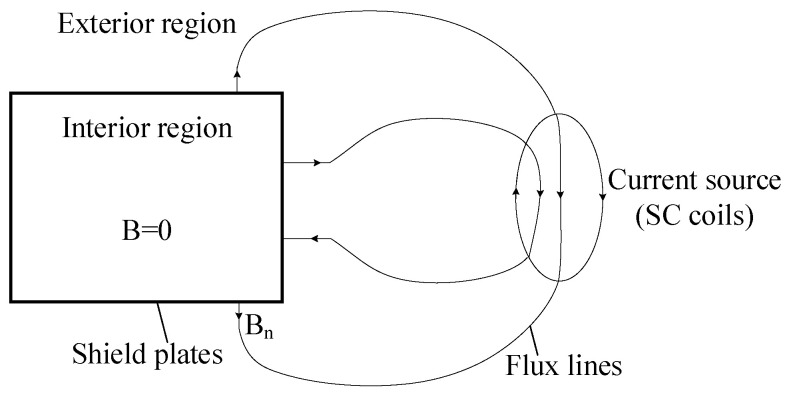
The structure of the exterior region and the interior region [23].

According to Assumption (1), the magnetic field from the current source in the external area enters the magnetic shielding plate vertically; according to Assumptions (1) and (2), there is no magnetic flux leakage into the internal area. The specific structure is shown in Figure 2.

Secondly, solving the electromagnetic balance equation and optimization problem of the magnetic shielding plate.

The Maxwell equation system is established for the static magnetic field in the external region:

(1) divB=0

Formula divB=0 defines a two-dimensional vector field ***F*** on a magnetic shielding surface, representing the magnitude and direction of magnetic flux. Assuming the magnetic shielding surface is a plane consisting of an *x* component, ***F****_x_*, and a *z* component, ***F****_z_*. The vector field satisfies the following expression:

(2) $$divF=B_{n}$$

Considering that the magnetic flux will not leak into the internal area, $B_{n}$ represents the magnetic flux density flowing vertically into or out of the shielding plate surface from the external area. Applying differential operations on a two-dimensional surface, Expression (1) can be equivalent to:

(3) $$\left\{\begin{array}{c} divB=0\\ divF=\partial (F_{x})/\partial x+\partial (F_{z})/\partial z=B_{n} \end{array}\right.$$

∂/∂x and ∂/∂z representing the average differential equations in the *x* and *z* directions.

On the magnetic shielding surface, Maxwell equations are established as:

(4) $$\left\{\begin{array}{c} divB=0\\ divF=\partial (F_{x})/\partial x+\partial (F_{z})/\partial z=B_{n} \end{array}\right.$$

The symbol B is the magnetic flux density generated by the SC coil, and the expression is:

(5) $$\left\{\begin{array}{c} B=c\sum_{m=0}^{\infty }\sum_{n=0}^{\infty }\frac{\sin(a_{m}a)}{a_{m}}\frac{\sin(a_{n}b)}{a_{n}}\exp(ja_{m}x+ja_{n}z-a_{mn}y)e\\ d=\frac{\mu_{0}H_{0}}{\mu H_{\mu }}R_{o} \end{array}\right.$$

where *m* and *n* are the Fourier decomposition coefficients, $a_{m}$, $a_{n}$ and $a_{mn}$ are functions related to Fourier coefficients, c is a function related to magnetic electromotive force, and a and b are the length and width of the SC magnetic source. *d* is shield thickness, $\mu_{0}$ is vacuum permeability, $H_{0}$ is knee point magnetic field strength, μ and $H_{\mu }$ are shield material permeability and magnetic field strength, $R_{0}$ is the distance from the magnetic source to the shield.

The optimization objective and its constraint expression are:

(6) $$\begin{array}{l} \min\int \int_{\partial \Omega }\left|F\right|dS\\ \mathrm{div}F=B_{n} \end{array}$$

It is assumed that $B_{n}$ is a constant value and ∂Ω is a set of bounded planes. On each plane, F can be decomposed $F_{x}$ and $F_{z}$.

An extended lagrange function expression is defined:

(7) $$L_{g}(F,\lambda )=\int \int_{\partial \Omega }L(F,\lambda )dS=\int \int_{\partial \Omega }\left|F\right|/B_{s}dS-\int \int_{\partial \Omega }\lambda (divF-B_{n})dS$$

where the λ is the lagrange multiplier, numerical problems are used for optimization. Firstly, the shield plate surface ∂Ω is divided into *N* sub-regions; secondly, the objective function is approximately calculated and processed. The optimization problem expression in step *k* can be obtained:

(8) $$\min\;\sum_{i=1,\cdots ,N}\iint_{\partial \Omega i}{\left|F\right|}^{2}/\left|F_{i}(k-1)\right|dS$$

The optimization problem is calculated by the mixed finite element method. Similarly, the lagrange multiplier is introduced, and the extended lagrange function expression is constructed:

(9) $${Lg}_{k}(F,\lambda )=\frac{1}{2}\sum_{i=1,\cdots ,N}\iint_{\partial \Omega }{\left|F\right|}^{2}/\left|F_{i}(k-1)\right|dS+\iint_{\partial \Omega }\lambda (divF-B_{n})dS$$

After integrating the second part of the above expression, the expression of the necessary conditions to meet the optimization can be obtained:

(10) $$\sum_{i=1,\cdots ,N}\iint_{\partial \Omega }\left(F,F^{\prime }\right)/\left|F_{i}(k-1)\right|dS+\iint_{\partial \Omega }(grad\;\lambda ,F^{\prime })dS=0$$

(11) $$\iint_{\partial \Omega }\left\{\left(F,grad\;\lambda^{\prime }+(\lambda^{\prime },B_{n})\right)\right\}dS=0$$

where the $F^{\prime }$ and $\lambda^{\prime }$ are test functions that can be freely selected from the same function space as F and λ. As a double integral $F_{i}(k)$ of the objective function, in the final step of solving $\partial \Omega_{i}$, the central value F(k) of the numerical solution is used, then the necessary conditions formulas are solved using the lagrange undetermined coefficient method.

**Figure 3:**
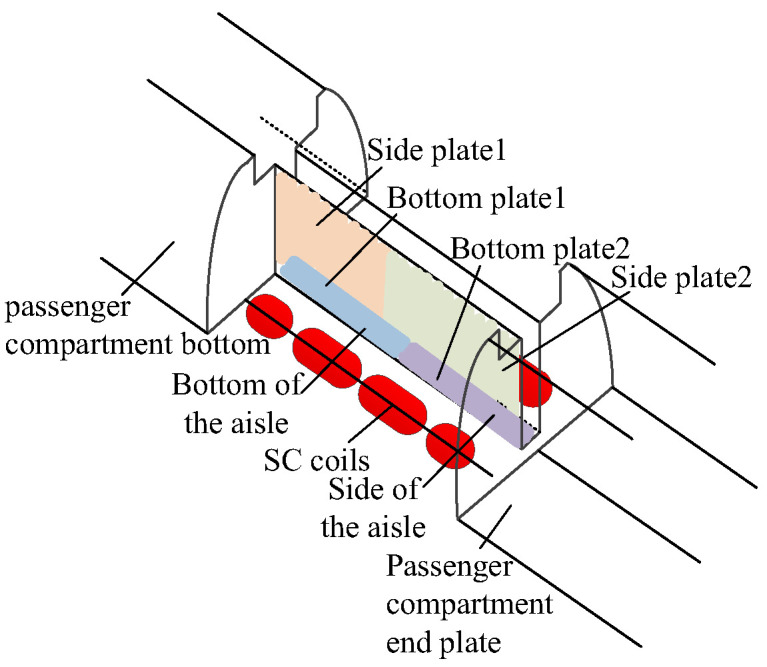
The structure of the optimized SC maglev train.

The 3D structure of the maglev train with optimized SC coils is shown in Figure 3. The optimized SC coils parameters are shown in Table 2. The analytical calculation process of SC coil structure optimization is compared by the extended lagrangian method and the Co-energy method, which can conclude that the method can effectively avoid the analytical error caused by the change in coil parameters in the co-energy method. The bottom and side plates of the magnetic shielding board are located at the bottom and side of the aisle. The shape of the magnetic shielding plate on each bogie is rectangular, with a weight of 697 kg, installed in the area around the articulated bogie between the two guest rooms, on the aisle, and guest room carriages.

## 4. Design and Analysis of Magnetic Shielding

The magnetic shielding effect in the aisle and the guest room, the magnetic flux density distribution at the magnetic shielding, is calculated and analyzed through analytical calculation.

### 4.1. The Calculation Analysis of Magnetic Flux at the Aisle and the Guest Room

**Figure 4:**
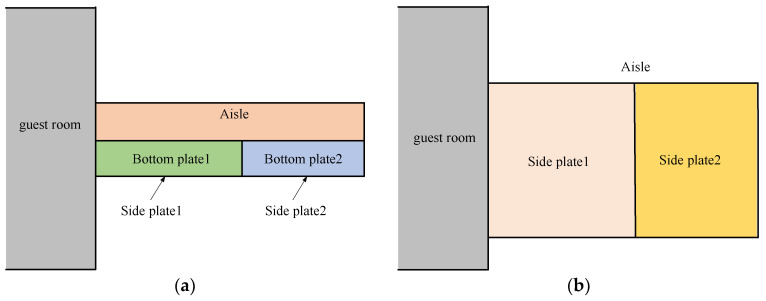
The position of bottom plate and side plate at aisle (**a**) vertical view, (**b**) side view.

**Figure 5:**
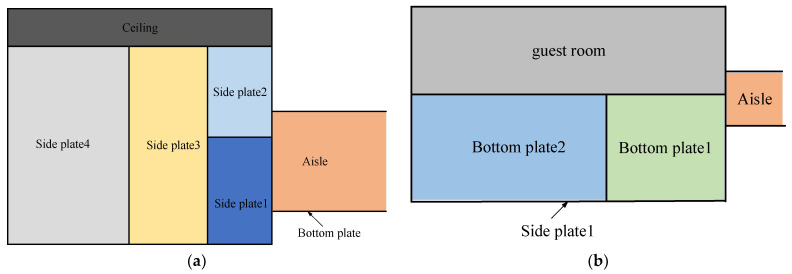
The position of bottom plate and side plate at guest room (**a**) vertical view, (**b**) side view.

SC coils are wound along the area surrounded by each rectangle shown by a thick line in Figure 4 and Figure 5. On the aisle, the position of the side plate and bottom plate is shown in Figure 4 from the perspective of a top view and a side view. In the guest room, the position of the side plate and bottom plate is shown in Figure 5 from the perspective of a top view and a side view.

**Figure 6:**
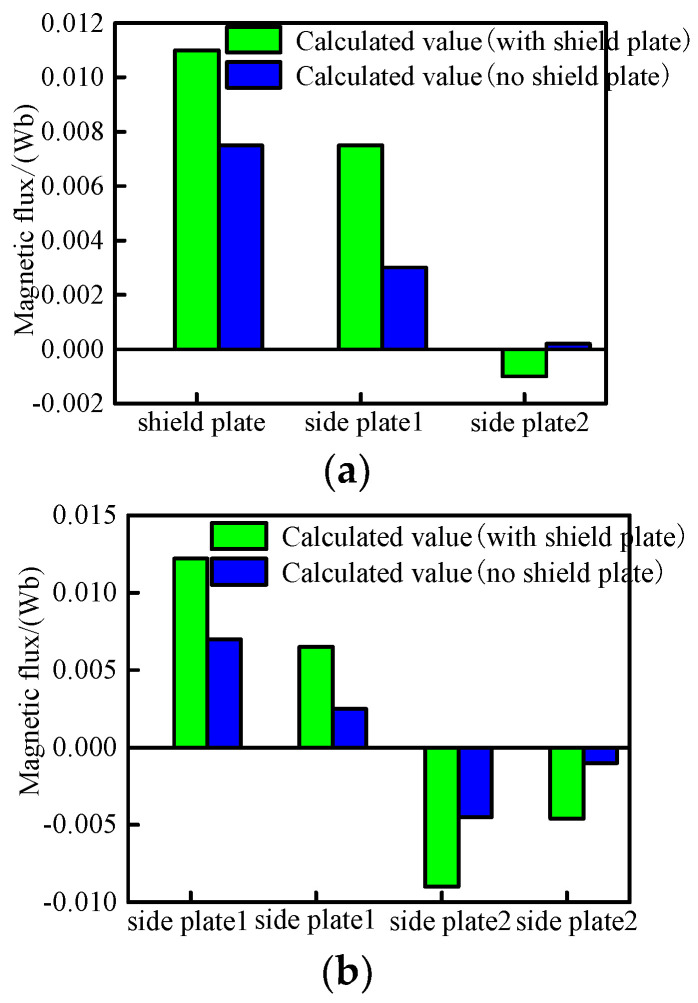

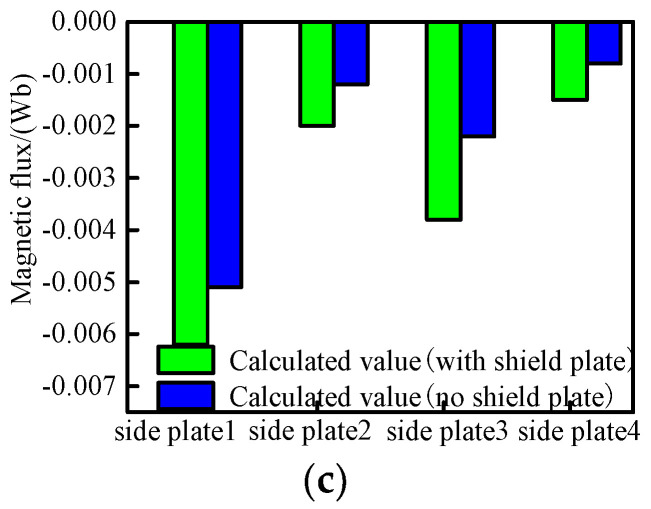
The calculated values of magnetic flux in aisle and guest room (**a**) The aisle connection, (**b**) The bottom and end plates of guest room, and (**c**) The side plates of guest room.

First, the magnetic shielding effect at the aisle is analyzed. The magnetic flux generated by the SC coils flows into or out of the bottom and side of the aisle. The calculation results of multiple locations are shown in Figure 6a. The magnetic flux enters the side plate one and the bottom plate one from the end SC coil N3, and then enters the SC coil S3 from the side plate two and the bottom plate two. The magnetic flux directions of the bottom plates one and two are opposite. The magnetic flux generated by the four internal SC coils (S_1_, N_2_, S_3_, N_4_) installed on each side is coupled with the adjacent coils, so there is almost no leakage magnetic flux to the magnetic shield. Next, the magnetic shielding effect in the guest room is analyzed. The calculation results of magnetic flux entering or exiting the guest room bottom and end plate are shown in Figure 6b, and the results in the side plate are shown in Figure 6c. With a magnetic shield, the magnetic flux density in the guest room is close to 1.1 mT; without a magnetic shield, the maximum magnetic flux density in the aisle is 14.3 mT, and the maximum magnetic flux density in the guest room is 25.3 mT. Therefore, the magnetic shield can insulate more magnetic flux from entering the guest room and aisle.

### 4.2. The Calculation Analysis of Magnetic Flux Density at Aisle and Guest Room

**Figure 7:**
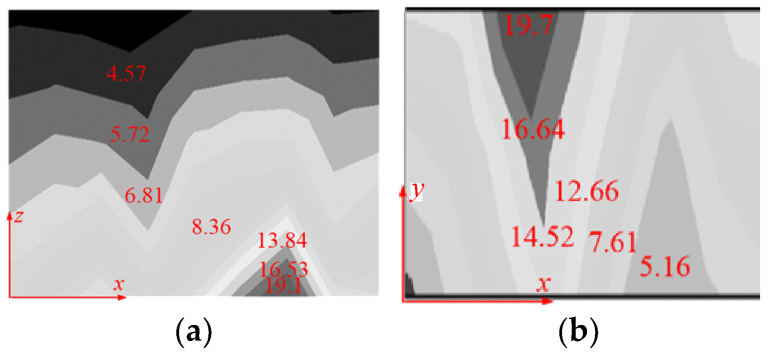
The magnetic flux density distribution map of the magnetic shielding plate at the aisle: (**a**) the side plate, (**b**) the bottom plate. (Unit: mT).

**Figure 8:**
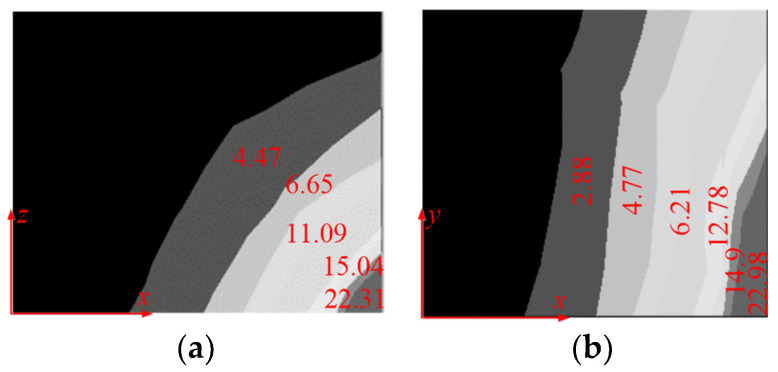
The magnetic flux density distribution map of the magnetic shielding plate in the guest room (**a**) the side plate, (**b**) the bottom plate. (Unit: mT).

The magnetic flux density distribution map of magnetic shielding plate at aisle and guest room by analytical calculations are shown in Figure 7 and Figure 8. The magnetic flux density distribution generated by the optimized SC coils is regular, and the magnetic flux density value at the corresponding position is reduced, which is significant for the weight decrease in the magnetic shield plate at the aisle. Compared with the existing structure, the magnetic flux density at the SC coils end of the structure is significantly reduced, and the area of the high magnetic flux density is significantly smaller, reducing the thickness of the magnetic shield plate.

**Figure 9:**
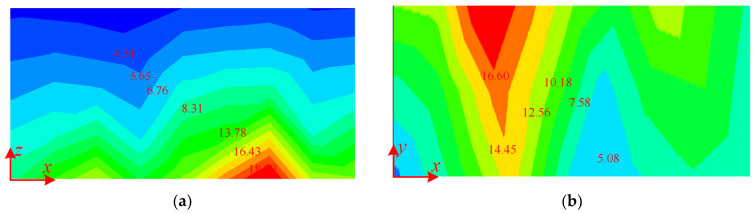
The magnetic flux density distribution map of magnetic shielding plate at aisle (**a**) The side plate, (**b**) The bottom plate. (Unit: mT).

**Figure 10:**
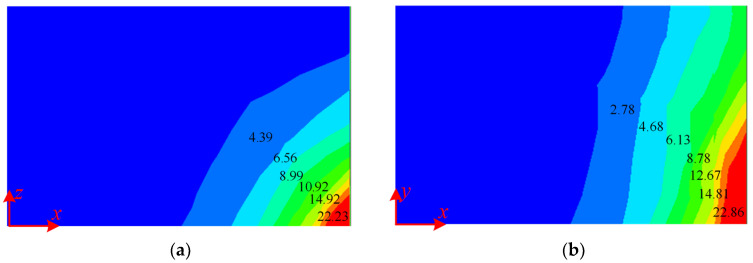
The magnetic flux density distribution map of magnetic shielding plate at guest room (**a**) The side plate, (**b**) The bottom plate. (Unit: mT).

The magnetic flux density distribution map of the magnetic shielding plate in the aisle and the guest room by FEM-based simulations is shown in Figure 9 and Figure 10. The comparison of magnetic flux density between analytical calculation and FEM results shows an error of less than 5%, which proves the accuracy of the analytical method above.

## 5. Conclusions

The magnetic shield characteristics of the SC maglev train are designed and analyzed by an analytical method. The magnetic shielding effect, magnetic flux density distribution are analyzed and calculated, relevant conclusions are obtained: (1) Whether in the guest room or aisle, the magnetic flux at the end of bottom plate and side plate is greater than those in the middle; (2) The magnetic flux density at the SC coils end is significantly reduced by optimization, and the area of the high magnetic flux density is significantly smaller; (3) The optimal magnetic flux density value is reduced by 50%, and the weight of the magnetic shielding plate is saved by 14%. There are still corresponding errors between the analytical calculations and finite element simulation values in this manuscript. In the future, when funds are sufficient, a high-speed maglev prototype model can be built, and the magnetic flux density distribution characteristics at corresponding positions can be tested to obtain more accurate values. The current analysis conclusions have certain guiding significance for the electromagnetic shielding characteristics of EDS high-speed maglev systems in practical engineering construction.

## Figures and Tables

**Table 1:** The partial structure and circuit parameters of SC coils.

SC Coils	Value
Number of coils	8
Polar distance (m)	1.35
Coil length (m)	1.07
Coil width (m)	0.50
Turns	1400

**Table 2:** The partial structure and circuit parameters of optimized SC coils.

SC Coils	Value
Number of coils	8
Polar distance (m)	1.05/1.5
Coil length (m)	0.8/1.25
Coil width (m)	0.50
Turns	2000

## Data Availability

All data generated or analyzed during this study are included in this published article.
